# Profile of Central and Effector Memory T Cells in the Progression of Chronic Human Chagas Disease

**DOI:** 10.1371/journal.pntd.0000512

**Published:** 2009-09-09

**Authors:** Jacqueline Araújo Fiuza, Ricardo Toshio Fujiwara, Juliana Assis Silva Gomes, Manoel Otávio das Costa Rocha, Ana Thereza Chaves, Fernanda Fortes de Araújo, Rafaelle Christine Gomes Fares, Andrea Teixeira-Carvalho, Olindo de Assis Martins-Filho, Guilherme Grossi Lopes Cançado, Rodrigo Correa-Oliveira

**Affiliations:** 1 Centro de Pesquisas René Rachou, FIOCRUZ, Belo Horizonte - Minas Gerais, Brazil; 2 Santa Casa de Misericórdia de BH, Pós-graduação em Biomedicina e Clínica Médica, Belo Horizonte - Minas Gerais, Brazil; 3 Departamento de Parasitologia, Instituto de Ciências Biológicas, Universidade Federal de Minas Gerais, Belo Horizonte - Minas Gerais, Brazil; 4 Faculdade de Medicina, Programa de Pós-graduação em Ciências da Saúde: Infectologia e Medicina Tropical, Universidade Federal de Minas Gerais, Belo Horizonte - Minas Gerais, Brazil; New York University School of Medicine, United States of America

## Abstract

**Background:**

Chronic Chagas disease presents several different clinical manifestations ranging from asymptomatic to severe cardiac and/or digestive clinical forms. Several studies have demonstrated that immunoregulatory mechanisms are important processes for the control of the intense immune activity observed in the chronic phase. T cells play a critical role in parasite specific and non-specific immune response elicited by the host against *Trypanosoma cruzi*. Specifically, memory T cells, which are basically classified as central and effector memory cells, might have a distinct migratory activity, role and function during the human Chagas disease.

**Methodology/Principal Findings:**

Based on the hypothesis that the disease severity in humans is correlated to the quality of immune responses against *T. cruzi*, we evaluated the memory profile of peripheral CD4^+^ and CD8^+^ T lymphocytes as well as its cytokine secretion before and after *in vitro* antigenic stimulation. We evaluated cellular response from non-infected individuals (NI), patients with indeterminate (IND) or cardiac (CARD) clinical forms of Chagas disease. The expression of CD45RA, CD45RO and CCR7 surface molecules was determined on CD4^+^ and CD8^+^ T lymphocytes; the pattern of intracellular cytokines (IFN-γ, IL-10) synthesized by naive and memory cells was determined by flow cytometry. Our results revealed that IND and CARD patients have relatively lower percentages of naive (CD45RA^high^) CD4^+^ and CD8^+^ T cells. However, statistical analysis of *ex-vivo* profiles of CD4^+^ T cells showed that IND have lower percentage of CD45RA^high^ in relation to non-infected individuals, but not in relation to CARD. Elevated percentages of memory (CD45RO^high^) CD4^+^ T cells were also demonstrated in infected individuals, although statistically significant differences were only observed between IND and NI groups. Furthermore, when we analyzed the profile of secreted cytokines, we observed that CARD patients presented a significantly higher percentage of CD8^+^CD45RA^high^ IFN-γ-producing cells in control cultures and after antigen pulsing with soluble epimastigote antigens.

**Conclusions:**

Based on a correlation between the frequency of IFN-γ producing CD8+ T cells in the T cell memory compartment and the chronic chagasic myocarditis, we propose that memory T cells can be involved in the induction of the development of the severe clinical forms of the Chagas disease by mechanisms modulated by IFN-γ. Furthermore, we showed that individuals from IND group presented more T_CM_ CD4^+^ T cells, which may induce a regulatory mechanism to protect the host against the exacerbated inflammatory response elicited by the infection.

## Author Summary

Chagas disease is a parasitic infection caused by protozoan *Trypanosoma cruzi* that affects approximately 11 million people in Latin America. The involvement of the host's immune response on the development of severe forms of Chagas disease has not been fully elucidated. Studies on the immune response against *T. cruzi* infection show that the immunoregulatory mechanisms are necessary to prevent the deleterious effect of excessive immune response stimulation and consequently the fatal outcome of the disease. A recall response against parasite antigens observed in *in vitro* peripheral blood cell culture clearly demonstrates that memory response is generated during infection. Memory T cells are heterogeneous and differ in both the ability to migrate and exert their effector function. This heterogeneity is reflected in the definition of central (T_CM_) and effector memory (T_EM_) T cells. Our results suggest that a balance between regulatory and effectors T cells may be important for the progression and development of the disease. Furthermore, the high percentage of central memory CD4^+^ T cells in indeterminate patients after stimulation suggests that these cells may modulate host's inflammatory response by controlling cell migration to tissues and their effector role during chronic phase of the disease.

## Introduction

Infection with the protozoan *Trypanosoma cruzi* is a major cause of morbidity and mortality in Central and South America, accounting for 12.500 deaths per year [1],[2]. The acute phase of infection is characterized by intense and evident blood parasitemia and may result in death. Upon infection, both the innate and adaptive immune responses lead to the control of parasite levels in the acute phase of the infection, but are insufficient for complete clearance of the *T. cruzi*. However, this phase is generally followed by an asymptomatic or indeterminate phase, during which there are no clinical symptoms or clear evidence of the presence of the parasite. The asymptomatic phase may last months to decades. Thus, most individuals are infected for life, with parasites persisting primarily in muscle cells, and approximately 30% of the individuals developing cardiac clinical form of Chagas disease [3],[4].

The scarce parasitism during the chronic phase, the prolonged latent period that precedes morbidity, and the intriguing existence of different clinical forms as well as a clear involvement of the immune response, have led several authors to evaluate the involvement of auto-immune factors in the pathogenesis of the disease. Some authors have pointed out the existence of cross-reactions between host tissues and *T. cruzi* antigens [5]–[14]. However, the demonstration of the presence of *T. cruzi* or its antigens by immunohistochemical techniques or of *T. cruzi* DNA by polymerase chain reaction (PCR) in inflamed myocardial tissues suggest that parasite antigens may be necessary to trigger the inflammatory process [15]–[17]. Therefore, both processes may be involved on the development of the severe clinical forms of the disease and may act synergistically as the disease progresses.

Although a significant percentage of the patients will develop the severe forms of the disease, a larger proportion remains asymptomatic throughout life. These observations have stimulated several investigators to study the processes involved on the development of severe pathology as well as on the maintenance of the asymptomatic forms of Chagas disease. In fact, many studies have demonstrated that immunoregulatory mechanisms are important for the control of infection, possibly affecting disease morbidity in chronic clinical forms [18]–[20]. We have recently observed that patients with the indeterminate (IND) clinical form of chronic Chagas disease have higher percentages of CD4^+^CD25^high^ T cell population secreting IL-10 expressing FOXP3 [18]. These data suggest that an increase in the secretion of IL-10 by regulatory T cells during the chronic phase of the disease may be associated with protection of the host against the severe pathology induced by type 1 immune response [18],[21],[22].

Immunity to *T. cruzi* is complex, minimally involving a substantial antibody response and the activation of appropriate CD4^+^ and CD8^+^ T cell responses. The role of CD8^+^ and CD4^+^ T lymphocytes in resistance to *T. cruzi* and in the severity of clinical disease remains unclear. In our laboratory, we have previously demonstrated that patients with the cardiac clinical form (CARD) of chronic Chagas disease elicit a robust immune response against the parasite, with high levels of IFN-γ and low levels of IL-10 [21],[22] and that this response is associated with severe cardiac pathology. Similarly, Abel et al. suggests that the ability to mount a vigorous IFN-γ-response may be associated with the development of cardiomyopathy [23]. However, Laucella et al. showed that patients with mild disease display substantial amounts of IFN-γ-producing T cells, suggesting that severe disease is prevented, rather than caused, by an immune response dominated by type-1 cytokine production [24]. Additionally, Garg and Tarleton demonstrated that enhancing type 1 immune responses through genetic immunization can substantially reduce the severity of disease in persistently infected mice (Garg and Tarleton, 2002).

Heterogeneity of the response is a hallmark of antigen-specific T cells. CD4^+^ T cells may develop into T helper cell 1 (TH1), TH2, or TH17 cells and likewise become antigen-specific regulatory cells [25]–[27]. According to the model proposed by Lanzavecchia and Sallusto [28], protective memory is mediated by effector memory T cells (T_EM_) that migrate to inflamed peripheral tissues and display immediate effector function, whereas reactive memory cell development is mediated by central memory T cells (T_CM_) that home to T cell areas of secondary lymphoid organs, have little or no effector function, but readily proliferate and differentiate into effector cells in response to antigenic stimulation. In fact, human T_CM_ are CD45RO memory cells that constitutively express CCR7 and CD62L, two receptors that are also characteristic of naive T cells, which are required for cell extravasation through high endothelial venules and migration to lymphoid organs [29]–[31]. Differently, human T_EM_ are memory cells that have lost the constitutive expression of CCR7, are heterogeneous for CD62L expression, and display characteristic sets of chemokine receptors and adhesion molecules that are required for homing to inflamed tissues [31].

In addition to the repertoire of cytokine secretion, effector CD4^+^ T cells exhibit diversity in the homing process, such as migration to peripheral nonlymphoid tissue and transit to lymph node follicles [32]. Furthermore, heterogeneity of CD8^+^ T cell effector gene expression has also been described [33], although it is not clear whether this represents physiologically distinct cell fates or simply fluctuation in activation state. Memory T cells are heterogeneous, with central memory cells patrolling secondary lymphoid tissues, recapitulating the surveillance of their naive progenitor, and effector memory cells acting as sentinels at frontline barriers [34]. Although the role and function of effector and memory subsets in protection or pathology and the nature of polarizing signals required for their differentiation are becoming increasingly clear, there are still outstanding questions that need to be addressed, which are mainly related to the mechanism of T cell fate. Many of these questions deal with fundamental uncertainties that are common to many areas of blood cell differentiation, such as the extent of fate diversity, the ontogeny and lineage relationship between opposing and kindred fates, and the degree of natural and therapeutic plasticity at different stages of differentiation. In the experimental mouse model, Martin and Tarleton [35] observed that antigen-specific CD4^+^ and CD8^+^ T cells maintain a T_EM_ phenotype during persistent *T. cruzi* infection. Interestingly, it was observed that T_CM_ CD8^+^ memory T cells can be generated and maintained despite pathogen persistence in *T. cruzi* infection. Furthermore, it was demonstrated that complete pathogen clearance through benzonidazole treatment results in stable, antigen-independent and protective T cell memory, despite the potentially exhausting effects of prior long-term exposure to antigen in chronic infection [36],[37]. On the other hand, Tzelepis and colleagues [38] showed that the differentiation and expansion of *T. cruzi*-specific CD8^+^ cytotoxic T cells (T_EM_ T cells) is dependent on parasite multiplication. In humans, an increase in total effector/memory CD8^+^ T cells (CD45RA^−^CCR7^−^) was observed in CARD patients [39]. However, the role of these subtypes of memory cells is still not completely understood in human Chagas disease. In the present study, we evaluated the profile of peripheral blood subsets of CD4^+^ and CD8^+^ T cells expressing naive/memory markers (CD45RA^high^/RO^high^), memory cell subtypes (T_CM_−CD45RO^high^CCR7^+^ and T_EM_− CD45RO^high^CCR7^−^) and production of cytokines by peripheral blood cells after *in vitro* stimulation with *T. cruzi* antigens to evaluate a possible relationship between the presence of these cells and the development of different clinical forms of the disease.

## Materials and Methods

### Recruitment of subjects

The initial cohort of study subjects was recruited 8 years ago at the Outpatient Referral Center for Chagas Disease of the Hospital das Clínicas, Federal University of Minas Gerais, Brazil. All study participants provided a written informed consent following the guidelines of the Ethics Committee of the Federal University of Minas Gerais. The study protocol complied with the regulations of the Brazilian National Council on Research in Humans and was approved by the Ethics Committee of the Federal University of Minas Gerais under the protocol COEP/UFMG-372/04. Individuals with systemic arterial hypertension, *diabetes mellitus*, thyroid dysfunction, renal insufficiency, chronic obstructive pulmonary disease, hydroelectrolytic disorders, alcoholism, previous clinical history suggesting coronary artery obstruction and rheumatic disease, or who were unable to fulfill the study requirements for annual examinations were excluded from the study. Individuals were considered seropositive for *T. cruzi* infection if two or more of the standard tests performed, indirect immunofluorescence, ELISA or indirect haemagglutination, were positive. Study participants were evaluated annually for a range of clinical and immunological parameters related to Chagas disease [22].

In this study, we investigated the immune response of 23 patients, who fulfilled the protocol described above, all in the chronic phase of the infection. The patients infected with *T. cruzi* were classified as being in the indeterminate phase of Chagas (IND) or having the cardiac (CARD) form of the disease as previously reported [18]. Individuals in the IND group (n = 9) ranged from 30 to 68 years of age. These individuals had no significant alterations in the electrocardiography, chest x-ray, echocardiogram, esophagogram and barium enema. The CARD group age (n = 14) ranged from 29 to 73 years, and presented echocardiographic and/or clinical and radiological signs of heart enlargement, with a final diastolic diameter of the left ventricle greater than 55 mm. The cardiac patients that participated in this study were classified as belonging to the group CARD V, as previously reported [18]. Twelve healthy individuals, 29 to 61 years old, from a non-endemic area for Chagas disease, and who had negative serology for Chagas disease, were included in the control group (NI).

### Antigens

Epimastigote (EPI) antigens were prepared by using the CL strain of *T. cruzi* as previously described [18],[21],[22]. Briefly, EPI were washed three times in cold phosphate-buffered saline (PBS), disrupted by repeated freezing at −70°C and thawing, homogenized at 4 to 6°C in a Potter-Elvejem (Vir Tis-Precise Wisconsin, USA) and centrifuged at 20,000×*g* five times at 4°C for 60 seconds, with 30 seconds intervals. The suspension was subsequently centrifuged at 40,000×*g* for 60 minutes in the cold. The clear supernatant was dialyzed for 24 hours at 4°C against PBS, filter sterilized on 0.22-µm pore-size membranes, assayed for protein concentration, aliquoted, and stored at −70°C until needed.

### Flow cytometric analysis of peripheral blood

Whole blood was collected in Vacutainer tubes containing EDTA (Becton Dickinson, USA) and 100 µL samples were mixed in tubes with 2 µL of undiluted monoclonal antibodies conjugated with fluorescein isothiocyanate (FITC), phycoerythrin (PE), R-phycoerythrin coupled to the cyanine dye Cy5™ (PE Cy5) or allophycocyanin (APC) for the following cell surface markers: CD4 (RPA-T4), CD8 (RPA-T8), CD62L (DREG56), CD45RA (HI100), CD45RO (UCHL-1), CCR7 (3D12) (all from BD Pharmingen, USA). After adding the antibodies, the cells were incubated in the dark for 30 minutes at room temperature.

Following incubation, erythrocytes were lysed using 2 mL of FACS Lysing Solution (BD Biosciences, USA) and washed twice with 2 mL of phosphate-buffered saline containing 0.01% sodium azide. The cells were then fixed in formaldehyde (4%) and permeabilized with saponin buffer (0.5%) (SIGMA, USA) for 15 minutes. After incubation, the cells were fixed in 200 µL of fixative solution (10 g/L paraformaldehyde, 1% sodium-cacodylate, 6.65 g/L sodium chloride, 0.01% sodium azide). Phenotypic analyses were performed by flow cytometry with a FACScalibur flow cytometer (BD Biosciences, USA). Data were collected on 1×10^5^ lymphocytes (gated by forward and side scatter) and analyzed using CellQuest software (BD Biosciences, USA).

### Flow cytometric analysis of cells culture

Whole blood was stimulated *in vitro* with EPI (25 µg/mL) antigens in RPMI 1640 media supplemented with 1.6% L-glutamine, 3% antibiotic-antimycotic, 5% of AB Rh-positive heat inactivated normal human serum, for 22 hours at 37°C and 5% CO_2_. Control cultures were maintained in culture media for the same period of time. During the last 4 hours of culture, Brefeldin A (SIGMA, St. Louis, MO, USA) (10 µg/mL) was added to the cultures [18]. Cultured cells were washed twice in PBS containing 1% bovine serum albumin and stained with monoclonal antibodies specific for the different cell-surface markers, as described above. The cells were then fixed in formaldehyde (4%) and permeabilized in saponin buffer (0.5%) for 15 minutes. Finally, the cells were incubated with monoclonal antibodies reactive to IL-10 (JES3-9D7) and IFN-γ (B27) (both from BD Pharmingen, USA). Phenotypic analyses were performed in a FACScalibur flow cytometer (BD Biosciences, USA), and data collected on 1×10^5^ lymphocytes and analyzed using the CellQuest software (BD Biosciences, USA).

### FACS analysis of surface markers and intracellular cytokine

Lymphocytes were analyzed for their intracellular cytokine expression patterns and frequencies as well as for cell surface markers using Cell Quest software. The frequency of cells was analyzed in four gates for each staining procedure: gate 1 (R1), lymphocyte gate (Suplementary Figure S1A); gate R2, T CD4^+^ and CD8^+^ expressing CD45RA^high^ and CD45RO^high^ lymphocytes (Suplementary Figure S1B) ; gate R3, T CD4^+^ and CD8^+^ lymphocytes (Suplementary Figure S1C); gate R4, T CD4^+^ and CD8^+^ lymphocytes expressing CD45RO^high^/CCR7^+^ (Suplementary Figure S1D); gate R5, T CD4^+^ and CD8^+^ CD45RA^high^/CD45RO^high^ lymphocytes secreting IFN-γ and IL-10 (Suplementary Figure S1E). Limits for the quadrant markers were always set based on negative populations and isotype controls.

### Statistical analysis

Analyses were performed using GraphPad Prism version 4.0 software (GraphPad Software Inc, USA). The nonparametric tests Kruskal-Wallis test was used to compare the three clinical groups (NI×IND×CARD), followed by Dunns test to compare all pairs of columns. Mann-Whitney nonparametric test was used to evaluate the significance of the cytokine production and compare the pairs of columns (NI×IND) (NI×CARD) (IND×CARD), comparing all Differences were considered significant when a p value of less than 0.05 was obtained.

## Results

### Individuals with the indeterminate form of Chagas disease have lower percentage of CD4^+^ and CD8^+^ naive (CD45RA^high^) and elevated percentages of memory (CD45RO^high^) T cells

**Figure 1:**
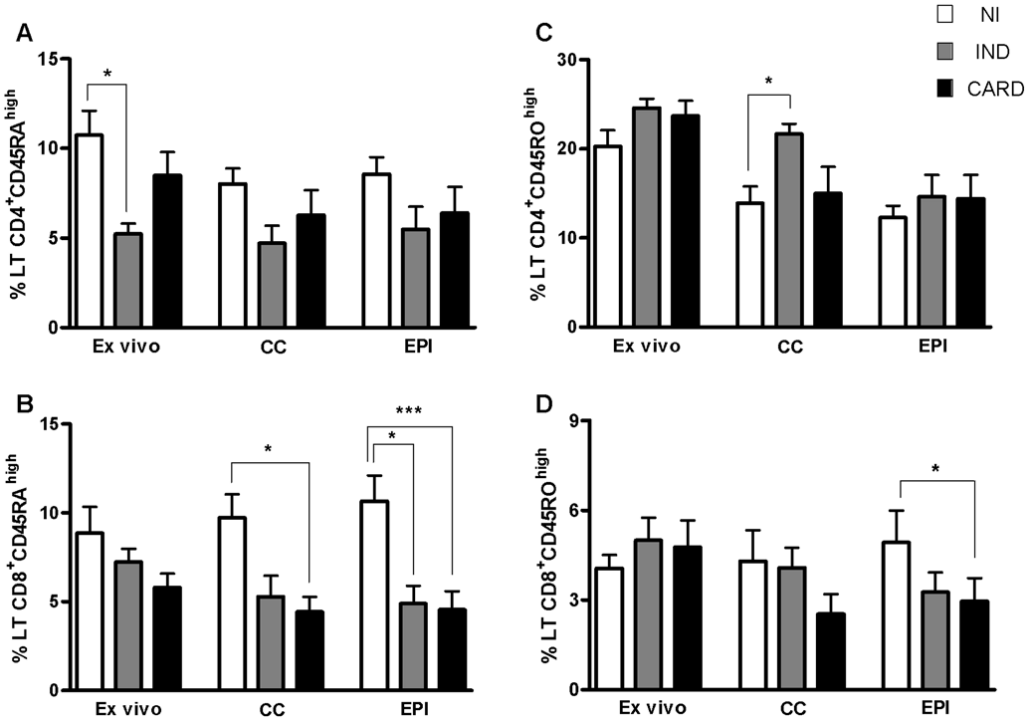
Naive and memory T cells. Percentage of CD4^+^ and CD8^+^ T cells, evaluated by flow cytometry before and after a short *in vitro* stimulation with *T.cruzi* antigen (EPI) as described in Material and Methods. A and B) CD45RA^high^ expression. C and D) CD45RO^high^ expression. *Ex vivo* = before stimulation, CC = culture non estimulated and EPI = culture after stimulation. The differences between the groups are considered significant at p less than 0.05 and are represented by * p<0.05, **p<0.01 and ***p<0.001.

The results show that the expression of CD45RA^high^ in CD4^+^ T cells, molecule expressed by naive T cells, was significantly lower (p<0.05) in IND patients when compared to NI individuals *ex vivo* (Figure 1A). We did not observe a significant difference between the infected groups when evaluated *ex vivo*. Furthermore, the percentage of CD8^+^CD45RA^high^ T cells in CARD patients, after culture in the absence of EPI antigens, was significantly lower (p<0.05) when compared to NI individuals (Figure 1B). Similarly, IND and CARD patients had a significantly lower percentage of CD8^+^CD45RA^high^ T cells, p<0.05 and p<0.01 respectively, when compared to NI individuals in *in vitro* cultures in the presence of EPI antigens (Figure 1B).

Our results also showed that the expression of CD45RO^high^ by CD4^+^ T cells, (memory lymphocytes), was not statistically different between NI, IND and CARD groups *ex vivo* and in culture of whole blood in the absence and presence of EPI antigens (Figure 1C and D). However, the percentage of CD45RO^high^ CD4^+^ T cells in the IND group was significantly higher (p<0.05) than in the NI group after culture without antigen stimulation (Figure 1C).

When we evaluated the expression of CD45RO^high^ by CD8^+^ T cells, the data did not reveal statistically significant differences between NI, IND and CARD groups, both *ex vivo* and in control cultures (Figure 1D). However, the CARD group showed a tendency to have higher percentages of these cells *ex vivo* when compared to the other groups (Figure 1D). Note worthy, after *in vitro* culture in the presence or not of EPI antigens, CARD patients presented a decrease in the percentage of these cells (Figure 1D).

### Chronic patients have an elevated percentage of T cells secreting cytokines

The presence of intracytoplasmatic cytokines IFN-γ and IL-10 in CD4^+^ and CD8^+^ T lymphocytes expressing either CD45RA^high^ or CD45RO^high^ surface markers was evaluated in NI, IND and CARD groups in the absence or presence of *in vitro* stimulation by EPI crude extract.

**Figure 2:**
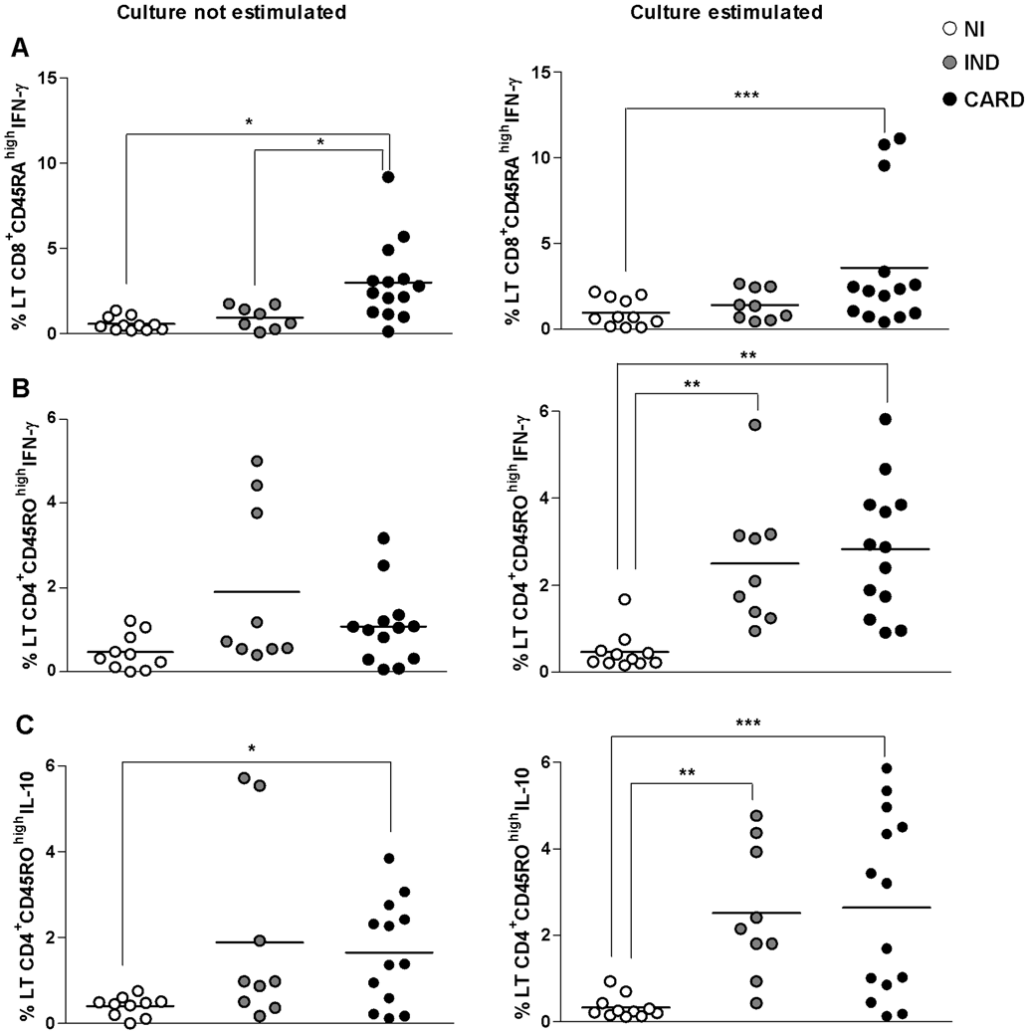
Cytokines secreted by naive and memory T cells. Percentage of CD8^+^CD45RA^high^, CD4^+^CD45RO^high^ and CD8^+^CD45RO^high^ T cells, evaluated by flow cytometry after a short *in vitro* stimulation *without* or with *T.cruzi* antigen (EPI) as described in Material and Methods. A and B) Intracellular cytokine IFN-γ expression. C) Intracellular cytokine IL-10 expression. CC = culture non estimulated and EPI = culture after stimulation. The differences between the groups are considered significant at p less than 0.05 and are represented by * p<0.05, **p<0.01 and ***p<0.001.

The data showed that CARD patients presented a significantly higher percentage (p<0.01 and p<0,001) of CD8^+^CD45RA^high^ IFN-γ-producing cells in control cultures (absence of antigen stimulation) when compared to IND patients and NI individuals. This percentage was also significantly higher in CARD group than in NI individuals (p<0.01) after antigen pulsing with EPI (Figure 2A). No significant differences were observed when we evaluated the percentage of CD8^+^CD45RA^high^ cells producing IL-10 in cultures in the absence or presence of EPI antigens (data not shown).

Data analysis revealed a significant increase (p<0.01) of IFN-γ^+^ CD4^+^CD45RO^high^ cells in whole blood samples from chagasic patients in comparison to NI individuals, after culture in the presence of EPI antigens (Figure 2B). There were no statistically significant differences among all studied groups evaluated after culture in the absence of antigenic stimulation (Figure 2B).

Analysis of IL-10^+^ CD4^+^CD45RO^high^ T cells showed a significant higher (p<0.05) percentage of these cells in CARD patients when compared to NI individuals after culture in the absence of antigenic stimulation (Figure 2C). The results showed that there is a significantly higher percentage of CD4^+^CD45RO^high^ T lymphocytes secreting IL-10 in CARD and IND patients (p<0.001 and p<0.01, respectively), after culture in the presence of antigens of EPI, when compared to NI group (Figure 2C).

Data analysis of the expression of cytokines IFN-γ and IL-10 by CD8^+^CD45RO^high^ T cells did not show any significant difference between the groups, after *in vitro* culture (data not shown).

### Identification of human CD4^+^ and CD8^+^ T cell memory subsets

Triple labeling of CD4^+^ and CD8^+^ T lymphocytes from peripheral blood with the molecules CD45RA, CD45RO and CCR7 allowed us to evaluate the process of recirculation of lymphocytes (percentage of cells migrating to/from secondary lymphoid organs).

We classified human CD4^+^ and CD8^+^ T cells by using two markers, CD45RO and CCR7. Three-color flow cytometry analysis demonstrated two major populations of human CD4^+^ and CD8^+^ T cells, i.e. CCR7^+^CD45RO^high^ and CCR7^−^CD45RO^high^.

**Figure 3:**
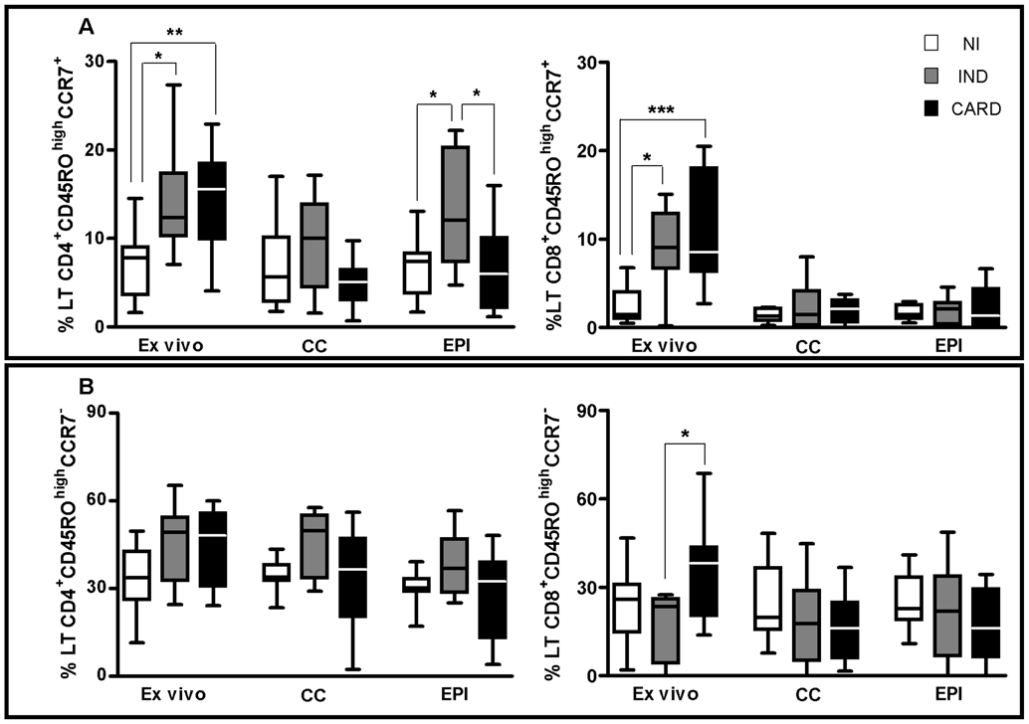
Analyses of central and effector memory T cells. Percentage of CD45RO^high^CCR7^+^ CD4^+^ and CD8^+^ T cells, evaluated by flow cytometry before and after a short *in vitro* stimulation with *T.cruzi* antigens (EPI) as described in Material and Methods. A) CD45RO^high^CCR7^+^ expression (T_CM_). B) CD45RO^high^CCR7^−^ expression (T_EM_). *Ex vivo* = before stimulation, CC = culture non estimulated and EPI = culture after stimulation. The differences between the groups are considered significant at p less than 0.05 and are represented by * p<0.05, **p<0.01 and ***p<0.001.

The assessment of the percentage of CD4^+^CD45RO^high^CCR7^+^ T cells demonstrated that CARD patients have significantly lower percentage (p<0.05) of this sub-population *ex vivo* when compared to NI individuals (Figure 3A). There were no significant differences in the analysis of these cells after culture (Figure 3A). In the analysis of CD4^+^ cells CD45RO^high^CCR7^−^, we did not find any differences on the percentage of cells between the groups, before or after stimulation (Figure 3B).

The expression of the CD45RO^high^CCR7^+^ phenotype by CD8^+^ cells, IND and CARD groups showed a significantly higher percentage of these cells (p<0.05 and p<0.001) in comparison to NI individuals in *ex vivo* (Figure 3A). The evaluation of these cells after culture did not show any statistically significant differences between the studied groups. (Figure 3A). When the percentage of CD8^+^CD45RO^high^CCR7^−^ T cells was assessed, we observed that CARD group presented a higher percentage (p<0.05) than the IND group *ex vivo*. A decrease on this percentage of this cell population was demonstrated after *in vitro* culture (Figure 3B). However, it was not statistically significant.

## Discussion

Most mature peripheral T cells are at rest and can be divided into naive and memory cells. This division is based on their response to antigens in a secondary response [40]. In addition to the functional activity, several markers have been identified to allow the distinction of these cell populations. Naive T cells, which have not encountered antigens, express high levels of CD45RA and L-selectin on its surface, and do not express activation markers such as CD25, CD44 and CD69 [40]. In contrast, memory T cells, which were previously stimulated by an antigen, express high levels of CD45RO and low levels of L-selectin [41]. In this study, we demonstrate that IND and CARD patients have less naive CD4^+^ and CD8^+^ T cells, demonstrated by decreased expression of CD45RA, as well as lower levels of CD8^+^ memory T cells when compared to NI (control) individuals. Similar results have been demonstrated by other study, in which patients in the chronic phase of Chagas disease presented the same expression profile of CD45RA^low^ in both CD4^+^ and CD8^+^ peripheral T cells [42]. Moreover, using an experimental model, another study demonstrated that splenic CD8^+^ T cells from *T. cruzi*-infected animals show lower expression of CD45RA than cells from non-infected mice [43]. The reduction of naive and increase of memory T cells may occur during the chronic phase of the disease. This might indicate a clonal T cell exhaustion due to continuous antigenic stimulation by persistent parasites, and may be associated with increased disease severity [42], [44]–[46]. The reduction in the expression of CD45RA is a direct result of the exchange of the CD45 isoform from A to C, possibly leading to an easier association with the TCR complex [47],[48]. The C isoform allows the cell to be activated by lower antigen stimulation and co-stimulation [49]. The evaluation of CD4^+^ T cells expressing CD45RO^high^ showed a higher percentage of these cells in IND group when compared to NI individuals after culture without antigenic stimulation. In fact, several studies have shown that altering the combination of CD45 isoforms dramatically affects immune function and disease severity in autoimmune models. Available data also show that this mechanism is related to an altered threshold for TCR signaling, altered cytokine production and response. This indicates that manipulating the patterns of CD45 expression or signaling pathways that it modulates might be a potential immunoregulatory strategy [47], [48], [50]–[52].

The mechanisms involved on the development of different clinical forms of Chagas disease are still poorly understood, suggesting that multiple factors may be involved in its establishment, such as cytokine production and profile of activation or differentiation status defined by subsets of memory [53]. The most studied cytokines in Chagas disease are IFN-γ and IL-10. Several studies have described some of the main cell populations involved in the production of these cytokines, and their relationship with pathology or regulation of immune responses during this infection [18], [22], [23], [54]–[60]. Our previous studies suggest a dual role for IFN-γ during human Chagas disease, which is observed during the different stages of the infection (acute and chronic phases) or in the presence or absence of treatment [22],[54]. It has been shown that during the acute phase of experimental mouse infection, IFN-γ participates in the elimination the parasite [55]. Furthermore, the protective role of IFN-γ has also been postulated in humans, as individuals submitted to treatment show a strong cellular response with secretion of high levels of this cytokine after *in vitro* stimulation of PBMC [54]. On the other hand, studies on human chronic Chagas disease have also shown that IFN-γ production may be harmful to the organism, since its unregulated production in the heart tissue may promote the destruction of the myocardium due to its cytotoxic effects. [23],[54],[61]. However, Laucella et al. showed a linkage between increased *T. cruzi*-specific T cell-mediated IFN-γ responses and decreased disease severity. [24]. Indeed, there are several important differences between all these studies, which might contribute to discrepancies in interpretation. Therefore, a long-term immunological follow-up of *T. cruzi*-infected patients may provide valuable information on the real role and contribution of IFN-γ responses to parasite control and disease development. In this paper, we evaluate the secretion of IFN-γ and IL-10 by CD4^+^ and CD8^+^ naive (CD45RA^high^) and memory (CD45RO^high^) T cells. We observed that the subjects from the CARD group presented higher percentage of naive CD8^+^ T cells secreting IFN-γ. This result suggests that naive CD8^+^ T cells might be associated with the development of the cardiac clinical form of the disease, probably as activated cells. Bourreau et al. [62], when evaluating the immune response of PBMCs from the NI individuals after *in vitro* stimulation with *L. guyanensis* antigens, demonstrated that CD8^+^CD45RA^+^ T cells producing IFN-γ are CD62L^−^CCR7^−^, while those producing IL-10 are CD4^+^CD45RA^+^CCR7^−^CD62L^+^ T cells, which are migrating to the inflammatory foci but are not yet activated. Our group has also shown that in Chagas disease, patients from CARD group develop a strong response against *T. cruzi* antigens, presenting high levels of IFN-γ and low levels of IL-10 [22]. Interestingly, here we observed that chronic patients have more CD4^+^CD45RO^high^ T cells secreting both IFN-γ and IL-10. Similarly, Antonelli *et al.* (2004) [63] demonstrated that PBMC from patients infected with *L. braziliensis* show high frequencies of memory CD4^+^ T cells producing both inflammatory (IFN-γ) and regulatory (IL-10) cytokines. This result suggests that these cells may not be the main source of these cytokines, since they are producing both IL-10 and IFN-γ cytokines. Therefore, they may not be so relevant on the development of the inflammatory process caused by protozoan infections.

Moreover, some studies have shown that memory T cells may be involved in the protection and development of Chagas disease [35]–[37],[39]. Nonetheless, a detailed evaluation of the cell populations involved in recall responses in human Chagas disease is still necessary. Memory T cells can be divided into two sub-populations, based on their heterogeneity, effector functions and response to the antigen or cytokines. Once activated, a fraction of primed T lymphocytes persists as circulating memory cells that can lead to protection and, upon secondary challenge, result in a qualitatively different and quantitatively enhanced response [31], [64]–[68]. The central memory (T_CM_) human T cells express CD45RO and also CCR7 and CD62L molecules, two important receptors related to the migration of T cells to peripheral lymphoid organs [30],[69]. When compared to naive T cells, T_CM_ have higher sensitivity to antigen stimulation, are less dependent on co-stimulation and provide efficient feedback for stimulation of dendritic and B cells. After evaluation of memory subtypes, we observed that chronic patients have more CD4^+^ and CD8^+^ T_CM_ cells *ex vivo*. However, after stimulation with EPI antigens, only the IND group showed more T_CM_ CD4^+^ T cells. These data suggest that when the overall T cell compartment is eventually driven to exhaustion, it exhibits a low frequency of competent parasite-specific CD8^+^ T cells and predisposes the subject for disease progression; this profile is presented by CARD patients with persistence of antigens or re-infection by *T. cruzi*. Recently, using the mouse model of *T. cruzi* infection, an increase in CD8^+^ T_CM_ cells was observed during long-term persistent infection [36]. Of note, these CD8^+^ T_CM_ cells were capable of antigen-independent survival after being transferred into naive mice, and were maintained despite the presence of persistent antigen stimulation. Similarly, Bustamante et al. documented the development of stable, antigen-independent CD8^+^ T cell memory after benzonidazole-induced cure of chronic infected mice [37].

It has been shown in mice that antigen-specific T cells maintain an effector memory phenotype (T_EM_ CD8^+^ T cells) during persistent *T. cruzi* infection [35],[38], suggesting that these cells may play an important role in the pathogenesis of the Chagas disease. However, the process of cell development and differentiation from naive into T_CM_ or T_EM_ is still not clearly understood. Studies with patients infected with HIV (human immunodeficiency virus) or LCMV (lymphocytic choriomeningitis virus) suggested that T cells go through the process of differentiation from **naive→T_CM_→T_EM_** [70],[71]. On the other hand, several authors suggest that the T_CM_ and T_EM_ cells are actually independent subpopulations which develop according to the biological environment, (eg.: presence of different cytokines or the anatomical region of activation) are independently maintained [71]–[73]. Finally, other studies on the infection LCMV T_CM_ show that the cells proliferate and/or convert into T_EM_ after re-exposure to the antigen, suggesting an alternative model of CD8^+^ T cell differentiation: **naive→T_EM_→T_CM_** [74]. However, our data suggest that the type of differentiation is **naive→T_CM_→T_EM_** [70],[75]. In fact, IND patients seem to have some regulation that prevents T_CM_ cells from becoming T_EM_, but further studies are still needed to elucidate this question. Previous studies have attempted to clarify the role of different subtypes of memory cells using the experimental mouse model and the CD45RA as a cell marker [39],[76]. In the current study, we evaluated the memory profile using CD45RO^high^CCR7^+^ phenotype as a marker for T_CM_ and CD45RO^high^CCR7^−^ for T_EM_ T cells.

In conclusion, our results showed that CARD patients have more naive CD8^+^ T cells secreting IFN-γ and T_EM_ CD8^+^ T cells than IND and NI groups. Based on a correlation between the frequency of IFN-γ producing CD8^+^ T cells in the T cell memory compartment and the chronic chagasic myocarditis, we propose that memory T cells might be involved in the induction of the development of the severe clinical forms of the Chagas disease by mechanisms modulated by IFN-γ. Conversely, some authors have demonstrated that high levels of IFN-γ are inversely correlated with disease severity [24],[39]. In this way, the role of IFN-γ in human Chagas disease progression is not clear and should be further elucidated by large follow-up studies. Moreover, we demonstrated that individuals from IND group presented higher levels of T_CM_ CD4^+^ T cells, which could induce immunoregulatory mechanisms to protect the host against the exacerbated inflammatory response elicited by *T. cruzi* infection. Studies of subtypes of immunological memory are still important for understanding the role of these cells in Chagas disease (immmunoregulatory or pathogenic). Therefore, additional longitudinal studies of changes in CD8^+^ T cell sub-populations in chronically infected subjects may reveal specific markers for progression to severe disease.

## Supporting Information

Figure S1 FACS analysis. (A) Identification of peripheral lymphocytes population from CARD patients in diagram of FSC×SSC. (B) Dot plot of FL-1×FL-4, displaying the frequency of CD4^+^CD45RO^+^ cells, after stimulation with EPI. The CD4^+^CD45RO^high^ (R2) populations were sorted using the indicated sorting gates. (C) Dot plot of FL-1×SSC, displaying the frequency of CD4^+^ (R3). (D) Dot plot of FL-4×FL-2, displaying the frequency of CD4^+^CD45RO^high^CCR7^+^ (UR). (E) Dot plot of FL-4×FL-2, displaying the frequency of CD4^+^CD45RO^high^ IFN-γ^+^ (UR).
(0.33 MB TIF)
