# Supplementary figures and images for: Profile of Central and Effector Memory T Cells in the Progression of Chronic Human Chagas Disease
Source: PLoS Negl Trop Dis. 2009 Sep 9;3(9):e512. doi: 10.1371/journal.pntd.0000512 (PMC2729721; doi:10.1371/journal.pntd.0000512)

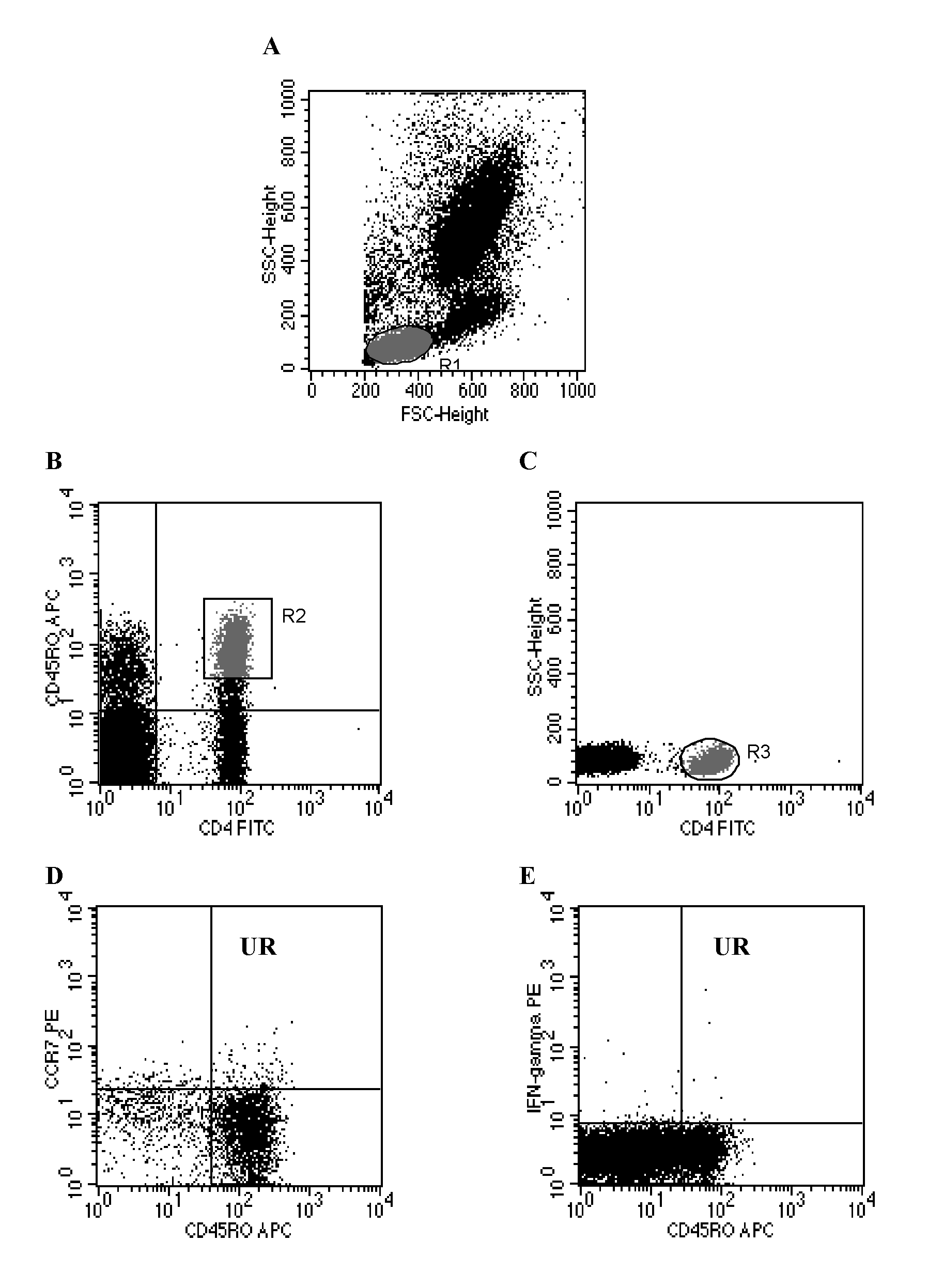

Supplement: Figure S1 — FACS analysis. (A) Identification of peripheral lymphocytes population from CARD patients in diagram of FSC×SSC. (B) Dot plot of FL-1×FL-4, displaying the frequency of CD4+CD45RO+ cells, after stimulation with EPI. The CD4+CD45ROhigh (R2) populations were sorted using the indicated sorting gates. (C) Dot plot of FL-1×SSC, displaying the frequency of CD4+ (R3). (D) Dot plot of FL-4×FL-2, displaying the frequency of CD4+CD45ROhighCCR7+ (UR). (E) Dot plot of FL-4×FL-2, displaying the frequency of CD4+CD45ROhigh IFN-γ+ (UR). (0.33 MB TIF) [file pntd.0000512.s001.tif]
